# Communicating Genetics and Smoking Through Social Media: Are We There Yet?

**DOI:** 10.2196/jmir.2653

**Published:** 2013-09-09

**Authors:** Sylviane de Viron, L Suzanne Suggs, Angela Brand, Herman Van Oyen

**Affiliations:** ^1^Operational Direction Public Health and SurveillanceScientific Institute of Public HealthBrusselsBelgium; ^2^Institute for Public Health Genomics (IPHG)Department of Genetics and Cell BiologyMaastricht UniversityMaastrichtNetherlands; ^3^Research Institute GROW (School for Oncology & Developmental Biology)Faculty of Health, Medicine and Life SciencesMaastricht UniversityMaastrichtNetherlands; ^4^Institute for Public Communication (ICP)Faculty of Communication ScienceUniversità della Svizzera italianaLuganoSwitzerland

**Keywords:** genetics, Internet, public health genomics, smoking, social media, Web 2.0

## Abstract

**Background:**

Social media is a recent source of health information that could disseminate new scientific research, such as the genetics of smoking.

**Objective:**

The objectives were (1) to evaluate the availability of genetic information about smoking on different social media platforms (ie, YouTube, Facebook, and Twitter) and (2) to assess the type and the content of the information displayed on the social media as well as the profile of people publishing this information.

**Methods:**

We screened posts on YouTube, Facebook, and Twitter with the terms “smoking” and “genetic” at two time points (September 18, 2012, and May 7, 2013). The first 100 posts were reviewed for each media for the time points. Google was searched during Time 2 as an indicator of available information on the Web and the other social media that discussed genetics and smoking. The source of information, the country of the publisher, characteristics of the posts, and content of the posts were extracted.

**Results:**

On YouTube, Facebook, and Twitter, 31, 0, and 84 posts, respectively, were included. Posts were mostly based on smoking-related diseases, referred to scientific publications, and were largely from the United States. From the Google search, most results were scientific databases. Six scientific publications referred to within the Google search were also retrieved on either YouTube or Twitter.

**Conclusions:**

Despite the importance of public understanding of smoking and genetics, and the high use of social media, little information on this topic is actually present on social media. Therefore, there is a need to monitor the information that is there and to evaluate the population’s understanding of the information related to genetics and smoking that is displayed on social media.

## Introduction

Social media are increasingly recognized as important tools for information provision, gathering, and transfer. They allow the spread of information to many people through different means [1]. For example, someone can publish a video on YouTube where anyone can view, listen, and even download the video. They can also create a group on Facebook to promote that video. In social media, every individual, regardless of credentials, is able to post and retrieve such information. Therefore, available information on social media is not exclusively based on experts’ knowledge but both on experts’ and laypersons’ experiences [2].

Health consumers use social media for a variety of purposes. A recent consumer survey observed that 24% of consumers posted information about their health experiences on social media platforms; 16% of consumers posted reviews of medications, treatments, doctors, or health insurers. Health symptoms or behaviors were traced and shared for 18% of the consumers. Health-related causes were joined by 20% of the consumers and supported by 28%. Furthermore, 16% of consumers share health-related videos or images on social media. Consumer trust in information posted on social media varied by messenger source ranging from hospitals (55%), from others they know (46%), health insurance companies (42%), and from unknown patients (25%). Regarding their susceptibility to share their own health information on social media, 30% would share this information with other patients, 43% with hospitals, and 38% with health insurance companies [2,3].

To date, most studies assessing the exposure to information about smoking on social media focused on pro- and anti-smoking information. Among adolescents, exposure to tobacco content appeared to be limited in volume, with 43% of adolescents being exposed to a mean of 13 pages of pro-tobacco content during 1-month follow-up [4]. The rate was nearly similar for anti-tobacco content, with 45% of the adolescents exposed to a mean of 10 pages of anti-tobacco content [4]. Some studies focused on the content of YouTube posts specifically, most of them being on tobacco control. But other topics were also developed: anti-smoking and quit-smoking posts as well as smoking-sexual fetish posts [2]. Facebook and Twitter are important ways to monitor the tobacco industry and to facilitate tobacco control [5]. As proposed by Hefler et al, social media strategies may be integrated into tobacco control organizations [5]. Moreover, social media, such as Facebook, already include information about many disorders and genetic syndromes [6].

Given the use of social media for health purposes and the increasing research, academic papers, and public and policy attention to genetic testing and genetic relationships with disease, it is expected that social media platforms are likely to become an important source for obtaining and disseminating genome-based information [7].

Despite the vast amount of research and efforts to prevent smoking and support cessation, smoking is still a major public health problem worldwide. Factors influencing smoking behavior are both nongenetic and genetic. Nongenetic factors included a broad range of aspects, such as social factors (eg, smoking status of peers), economic factors (eg, level of income), or psychological factors (eg, weight concerns). For genetic factors, the two main factors are the genes influencing the nicotine metabolism and genes influencing the cascade theory of reward [8].

Genetics and smoking are highly covered topics on different media. For example, a search for “genetics” and “smoking” on PubMed (including scientifically based content) resulted in 15,948 results. A search on Google (including both scientifically and nonscientifically based content) revealed 9,970,000 hits. On YouTube, 1,300,000 posts were obtained, on Facebook 472,000, and 8020 on Twitter (using the searches “smoking + genetic + site:YouTube.com”, “smoking + genetic + site:facebook.com”, and “smoking + genetic + site:twitter.com”). We also conducted a search of a specialized social media platform, PatientsLikeMe, and found 167 hits. However, upon review of the posts, only one publication about chronic obstructive pulmonary disease (COPD) and genetics was listed (and this publication was displayed multiple times). The high number of results in both scientific and nonscientific search sources suggests that such information could also be available in popular social media and could be found by lay public using typical simple search strategies.

Given the reach of social media and the growing reliance on it for health purposes, combined with the importance of genetics and smoking, this study aims to explore the availability of genome-based information about smoking on three popular social media platforms (YouTube, Facebook, and Twitter). Questions examined include (1) What type of information about genetics and smoking is displayed on social media?, (2) What is the source (scientific or nonscientific) of the posted information?, (3) What is the role of the publisher?, and (4) What countries is information being posted from? We expected that the information would be posted primarily from scientific sources from research centers in the United States and Europe.

## Methods

### Sample

Posts from YouTube, Facebook, and Twitter were included. YouTube (video sharing), Facebook (social networking), and Twitter (social networking and microblogging) are each ranked among the top 10 most popular websites [9]; Facebook was positioned second place just after Google. YouTube and Twitter were respectively at positions 3 and 8. The other websites included in the top 10 were retail websites (eg, Amazon.com) or Web search engines (eg, Google). Therefore, YouTube, Facebook, and Twitter appeared to be the most relevant social media. The number of users on social media is growing daily; however, Facebook is still the top-used medium with around 1.01 billion active users in 2012 [10]. Twitter counts roughly 500 million users and 200 million active users [11], and over 800 million unique users visit YouTube [12] each month.

### Search Strategy

**Figure 1:**
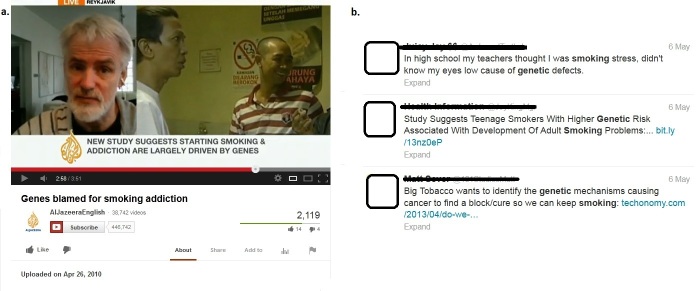
Screenshots of YouTube and Twitter results. (a) screenshot of YouTube; (b) screenshot of Twitter.

We searched for the terms “genetic” and “smoking” in each of the three social media. These terms were selected because they were simple terms that the general population may use to get information on the topic. The search was performed using two time points: the first one on September 18, 2012, and the second on May 7, 2013. The first 100 posts available for each social media platform were examined. Posts on YouTube were searched with the “relevance” option (the default option). On Facebook, posts were searched using both the total results and results by “people”, “pages”, “groups”, “apps”, “events”, “music”, “public posts”, and “posts in groups”. On Twitter, only posts of the previous days (approximately 1 week depending on the storage capacity of Twitter’s database) were visible at Time 1. This changed at Time 2, when posts from the previous months were available. Posts were excluded if they did not express the link between genetics and smoking tobacco (including smoking initiation, addiction, cessation, and smoking-related diseases). Figure 1 shows an example of the posts retrieved from YouTube and Twitter.

The first 100 posts were searched on Google at Time 2 using the same search terms. The aim of searching the posts on Google was twofold: first, to indicate the type of information available on the Internet and second, to determine if there were other types of social media that discussed genetics and smoking (eg, health forums or blogs).

### Data Extraction

From the different posts, the date of the publication and the country of the publisher were extracted. To understand, at least partially, the credibility of the information provider, the role of the publisher was coded and classified as a research center, news, medical news, independent user (ie, the person posting on social media was acting as an individual citizen and not on behalf of a group of people or organization or as a scientist), and other, if it belonged to none of the previous categories (eg, foundation such as “Arthritis Foundation” or companies such as “23andme”). The source of posted information was categorized as “scientific publication”, “referring to a scientific publication”, and “not referring to a scientific publication”. This allowed us to understand the differences in types of content posted on the three social media. The content of the posts was classified into smoking initiation, addiction, cessation, and smoking-related diseases. At least a link between genetics and the specific category must have been mentioned to allow the classification. Moreover, one post may have been classified in more than one category. For smoking-related diseases, we extracted the disease of interest. When available, we extracted the number of views and the opinion (like or dislike) of the post.

### Statistical Analyses

Univariate analyses were assessed by Pearson chi-square for categorical data and the Kruskal-Wallis one-way analysis of variance for continuous data (because the continuous variables were not normally distributed). Tests were two-sided with a significance rate of alpha=0.05. Tests were corrected for multiple testing through Bonferroni-Sidak. *P* values less than .001 remained significant after correction for multiple testing. All statistical analyses were performed using Stata, version 10.1.

To show the content of the posts on Twitter, the titles of the posts on YouTube, and the most frequent words found in the titles of the Google search, word clouds were used. Word clouds visually represent the frequency of the words used in the posts with larger size for more frequent words. Word clouds were created with the “wordcloud” package using the R project for statistical computing (R, version 2.14.1).

## Results

### Characteristics of the Three Social Media

**Figure 2:**
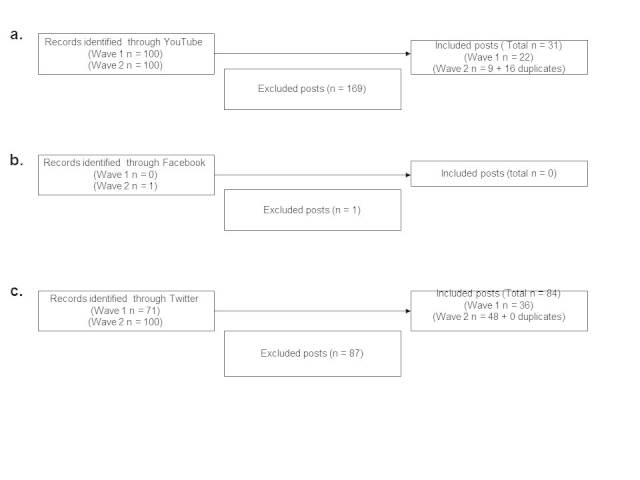
Flowcharts of the post selection: YouTube, Facebook, and Twitter. (a) YouTube flow chart; (b) Facebook flow chart; (c) Twitter flow chart.

Across both data collection points, YouTube, Facebook, and Twitter retrieved a total of 200, 0, and 171 posts respectively. Among those, 31, 0, and 84 discussed the genetics of smoking. On YouTube, 16 posts were retrieved both at Time 1 and 2. Moreover, from the 9 posts selected at Time 2, three were published after Time 1. By contrast, Time 1 (September 2012) and 2 (January to May 2013) did not overlap on Twitter. Therefore, no posts were found at both data collection points in Twitter (Figure 2). The number of included and excluded posts was significantly different between the three different social media (*P*<.001). Twitter obtained a higher proportion of posts discussing genetics and smoking (49.1%, 84/171) in comparison to YouTube (15.5%, 31/200).

When comparing included posts obtained from Twitter and YouTube (Table 1), no differences in the source of information or in the country of the publisher were observed. However, the role of the publisher was significantly different between the two media (*P*<.001). Most publishers were independent users on Twitter (45.2%, 38/84), although it was the smallest role category (3.2%, 1/31) on YouTube. On YouTube, most posts were published by news or medical news instead of independent users on Twitter. For the content of the posts, a higher number of YouTube posts reported an impact of genetics on smoking-related disease than on Twitter (*P*=.001). The other contents (smoking initiation, addiction, and cessation) obtained similar results on YouTube and Twitter. For smoking-related diseases, no comparison of the different types of disorders led to differences between YouTube and Twitter.

Between the two time points for both YouTube and Twitter, posts did not differ in the source of information, role of the publisher, country of the publisher, and characteristics of the posts. On YouTube, the content of the posts were not different between the two time points. By contrast, on Twitter, the content differed for smoking initiation (*P*<.001), addiction (*P*<.001), cessation (*P*<.001), and related disease (*P*<.001) (Table 2). Moreover, on Twitter, there was a significant difference in the number of days the posts were available (*P*<.001).

### Comparison Between Social Media and Google Search

Of the first 100 websites retrieved from the Google search, 86 were related to genetics and smoking. No new social media channels were revealed from this search. Websites retrieved from Google search were different from the posts on YouTube and Twitter, both in source of information and role of publisher. On Google, websites were more often scientific publications (46.5%) than on YouTube (0.0%) or Twitter (5.9%), explaining also the difference in the role of the publisher (scientific database, 46.5%) (Table 1).

Some scientific publications referred to on YouTube were also found in the Google search (eg, Amos et al [13] was listed 5 times on YouTube and once on Google) and the same for Twitter (eg, Govidan et al [14] appeared 12 times on Twitter and 1 time on Google, and Belsky et al [15], 38 times on Twitter and 9 on Google). However, no scientific publication found on YouTube was also retrieved on Twitter.

### Word Clouds of the Three Social Media and Google Search

**Figure 3:**
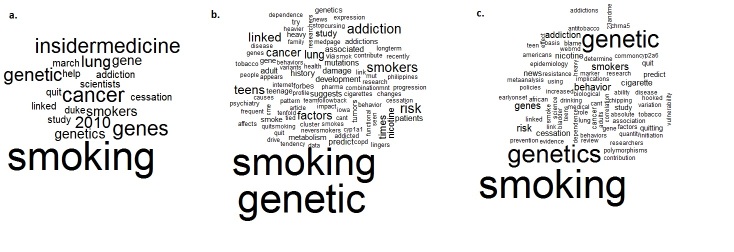
Word clouds presenting the most used words on YouTube, Twitter, and Google. (a) word cloud including the most used words in the title of YouTube posts; (b) the most used words from the posts in Twitter; (c) the most used words in Google. Each word’s frequency is correlated with font size.

Further exploration of the Twitter posts and the post titles on YouTube and Google search through word clouds (see Figure 3; frequency of words correlates to size of font) showed that the words “smoking”, “genetic”, and “cancer” were highly present. This result was in line with the high level of posts assessing smoking-related diseases. On YouTube, the word “Insidermedicine” was also highly reported. Over the 31 included posts, 6 were from Insidermedicine and summarized new studies published in scientific journals. The “2010” word in the graph is also due to Insidermedicine posts where the date of publication was written in the title. On Twitter, among others, the words “lung”, “addiction”, and “teens” were frequently reported. This referred to two scientific publications that were reported multiple times; 12 Twitter posts (14.3%) reported that smokers with lung cancer have tenfold genetic damage in comparison to never-smokers [14], and 38 posts (45.2%) referred to the genetic factors influencing addiction in teens [15]. In the Google search, the words “addiction”, “cessation”, and “risk” were most often used, giving an indication of the content of the websites.

**Table 1:** Characteristics of posts from YouTube and Twitter (*P* values from Pearson chi-square).

Variables		YouTube (n=31)	Twitter (n=84)	Google (n=86)	*P* value (YouTube vs Twitter)	*P* value (YouTube vs Twitter vs Google)
**Source of information, n (%)**					0.19	<.001^a,b^
	Scientific publication	0 (0.0)	5 (6.0)	40 (46.5)		
	Referring to a scientific publication	30 (96.8)	71 (84.5)	46 (53.5)		
	Not referring to a scientific publication	1 (3.2)	8 (9.5)	0 (0.00)		
**Role of the publisher, n (%)**					<.001^a,b^	<.001^a,b^
	Research center	8 (25.8)	4 (4.7)	4 (4.7)		
	News	11 (35.5)	7 (8.3)	16 (18.6)		
	Medical news	10 (32.3)	17 (20.2)	19 (22.1)		
	Independent user	1 (3.2)	38 (45.2)	0 (0.0)		
	Scientific database	0 (0.0)	0 (0.0)	40 (46.5)		
	Other	1 (3.2)	18 (21.4)	7 (29.9)		
**Country of the publisher, n (%)** ^c^					0.18	.003^b^
	United States	21 (70.0)	42 (50.6)	61 (81.3)		
	United Kingdom	2 (6.7)	1 (1.2)	7 (9.3)		
	Canada	1 (3.3)	3 (3.6)	0 (0.0)		
	Italy	0 (0.0)	3 (3.6)	1 (1.3)		
	Other	2 (6.5)	14 (16.7)	15 (17.4)		
**Characteristics of the post, median [percentile]** ^d^						
	Total # of days available	876 [319; 1441]	12.5 [5; 39]	707 [229.5; 1950.5]	<.001^a,b^	<.001^a,b^
	Duration (min)	1.61 [1.43; 2.77]	—	—	—	—
	Total number of viewership	232 [64; 1037]	—	—	—	—
	Total number of likes for the post	1 [0; 2]	—	—	—	—
	Total number of dislikes for the post	0 [0; 1]	—	—	—	—
**Content of the post, n (%)**						
	Smoking initiation	2 (6.5)	15 (18.1)	17 (19.8)	0.12	0.23
	Smoking addiction	14 (45.2)	53 (63.9)	62 (72.1)	0.07	0.03^b^
	Smoking cessation	8 (25.8)	23 (27.7)	32 (37.2)	0.84	0.31
	Smoking-related diseases	21 (67.7)	29 (34.5)	34 (39.5)	0.001^a,b^	0.005^b^
**Type of smoking-related diseases, n (%)** ^e^						
	Lung disease	1 (4.8)	0 (0.0)	0 (0.0)	0.24	0.22
	COPD	2 (9.5)	5 (17.2)	1 (3.0)	0.44	0.17
	Lung cancer	12 (57.1)	16 (55.2)	21 (63.6)	0.89	0.78
	Cancer in general	4 (19.1)	3 (10.3)	3 (9.1)	0.38	0.52
	Cardiovascular disease	1 (4.8)	1 (3.5)	1 (3.0)	0.82	0.95
	Multiple diseases	0 (0.0)	1 (3.5)	3 (9.1)	0.39	0.29

^a^Significant *P* values after Bonferroni-Sidak correction for multiple testing.

^b^Significant *P* values.

^c^On YouTube, there were 5 missing values, 21 on Twitter, and 2 on Google search.

^d^Median values with percentiles [p25; p75] and *P* value from Kruskal-Wallis one-way analysis of variance.

^e^Only posts referring to smoking-related diseases were used; COPD—chronic obstructive pulmonary disease.

**Table 2:** Comparison of the posts’ content between Time 1 and 2 (*P* values from Pearson chi-square).

	YouTube			Twitter		
	Time 1	Time 2	*P* value	Time 1	Time 2	*P* value
Smoking initiation, n (%)	0 (0.0)	2 (22.2)	.02^a^	0 (0.0)	15 (31.9)	<.001^a,b^
Smoking addiction, n (%)	8 (36.4)	6 (66.7)	.12	13 (36.1)	40 (85.1)	<.001^a,b^
Smoking cessation, n (%)	6 (27.3)	2 (22.2)	.77	1 (2.8)	22 (46.8)	<.001^a,b^
Smoking-related disease, n (%)	17 (77.3)	4 (44.4)	.08	22 (61.1)	7 (14.6)	<.001^a,b^

^a^Significant *P* values.

^b^Significant *P* values after Bonferroni-Sidak correction for multiple testing.

## Discussion

### Principal Findings

The current study introduced the availability of information regarding genetics and smoking in three different social media (YouTube, Facebook, and Twitter). This is, to our knowledge, the first study that investigated the availability of genetics and smoking in social media.

The results indicated that little information is available regarding the topic and even less on Facebook. The lack of posts on Facebook was not surprising given the specialized topic. However, we do expect the number of posts to grow dramatically as genetic testing and personalized medicine become more widely known. For YouTube and Twitter, most posts referred to scientific publications that were recently published.

The reason why no posts on genetics and smoking were retrieved using Facebook, versus the 472,000 posts obtained by using the search “smoking + genetic + site:facebook.com”, is that Facebook is not a search engine and Google is. Interestingly, the same search strategy in another search engine resulted in a different number of retrieved posts (eg, 37,000 posts were found using Bing). Nonetheless, social media do not allow for such advanced search strategies and typical users do not typically use such search strings. Our objective was to know what information lay people find on genetics and smoking when using social media, so we developed our search within those social media and used typical search strategies.

The reason for the high number of posts coming from the United States in comparison to other countries may be explained by the size of the country and high Web adoption rate [16], rather than the importance of the topic in any country. For example, Facebook had the highest number of users in the United States (167,554,700 users). Brazil was second with 60,665,740 users. In comparison, the United Kingdom had 33,227,180 users and was in sixth position [17]. Despite the European ITFoM project (IT Future of Medicine) [18], PHGEN project (Public Health Genomics European Network) [19], the activities of the EAPM (European Alliance for Personalised Medicine) [20], and the significant attention to it at the policy level in Europe, the mere size of the continents and way in which each public accesses science information likely explain why the European posts were fewer than in the United States.

On Twitter, the period of availability of the posts changed between Time 1 and 2 (from approximately 1 week to several months). This may explain the highest number posts included in Time 2.

The contents of the posts mostly focused on genetics of addiction and smoking-related diseases. This result is consistent with the higher number of scientific publications on both topics than on any other topic linking genetics and smoking. The smoking-related disease of interest was mainly lung cancer; this is also in line with the knowledge of the general population. Simon et al reported that smoking as a risk factor for lung cancer was recalled by 85% of the population and recognized in a list of factors by 94% of the population [21].

The opinion about the posts on YouTube (measured by the number of likes and dislikes, as reported in Table 1) should be viewed cautiously due to the small number of observations. The range of “like” posts was between 0 and 16 and “dislike” between 0 and 4, while the number of viewers ranged from 17 to 10,350. Moreover, for the posts that were retrieved in both time points, the number of likes and dislikes did not change significantly.

The difference between YouTube and Twitter for the number of days of a post’s availability on the Web (*P*<.001) is explained by the fact that the Twitter search was performed by date and that posts on YouTube were classified by relevance. The search by date for Twitter and relevance for YouTube were used because they were the default options and therefore, probably the most frequently used by the population. Nevertheless, no differences between YouTube and Twitter were reported in the source of information and the country of the publisher, making the information comparable.

When using Google search as an indicator of the information about genetics and smoking available on the Web, we first observed differences between the posts retrieved on social media. As we expected, most websites were scientific publications (46.5%) or referred to scientific publications (53.5%). Six scientific publications were referred at least once in the Google search and either on YouTube or Twitter [13-15,22-24]. The second objective of searching on Google was to discover any other social media that included discussions of genetics and smoking. Despite our expectations, no social media, such as health forums or blogs, were retrieved. This might be explained by the novelty of the topic.

Information on genetic testing and smoking is already on the Web with direct-to-consumer testing such as 23andMe where they look for the *CHRNA3* gene, more specifically the variant rs1051730. *CHRNA3* is a nicotinic receptor. Tests on smoking-related disorders are also available on 23andMe for lung cancer. As proposed by Pray in 2008, “imagine reading this warning on a cigarette package: Smokers with a particular mutation have a dramatically higher risk of developing lung cancer. Would you get tested for this mutation?” [25]. In the future, this kind of message may also be displayed on social media.

The way that individuals understand the posts on YouTube and Twitter should be assessed in a further study. Indeed, it is becoming more common that people are looking to different social media to get information about their health [3]. As public health genomics, personalized medicine, and personalized health terms become more commonly known terms, the public, including the general population, patients, and health professionals, will certainly look to social media to learn more about them and discuss them. Hence, information must be translated and communicated in a way for the general public to understand, especially since genome-based information, which includes genetic information, is a complicated topic for the nonscientific population.

Providing health information via social media and developing methods to evaluate their impact may help in effectively increasing health literacy and risk awareness in an innovative way, with attention to avoiding the introduction or widening of health inequalities. However, to generate effective health information messages, different conditions may be needed depending, for example, on the topic, target group, or society, taking into account both environment (internal and external) and the process of information (automatic and rational) [26]. Therefore, there is a real need to develop efficient communication tools to improve the health and genetic literacy of the population. Moreover, any message should aim to be correct, clear, and adapted to the target population to maximize understanding of the content. Also, any ethical and legal issues of displaying such messages should be considered. Achieving these conditions is of critical importance to develop quality information that can be obtained and understood by those accessing it and those who need it.

Future research should examine the impact that information about genetics and smoking on popular social media has on target population literacy and behaviors. Exposure to genetic information about smoking in social media might be examined in various target populations (eg, university students and pregnant women) in controlled settings where the target population is exposed to different genetic information about smoking during a certain period of time (eg, 1 week). After the exposure step, the impact of the information and information channel on different outcomes (eg, behavior change, genetics and smoking knowledge) would be assessed. The exposure-outcome relationship might then be evaluated using advanced statistical methods, such as structural equation modeling. Finally, as with any communication channel, content spread through social media channels should be carefully scrutinized by the reader. All media have the potential to include biased and misleading information, but social media platforms can spread such information rapidly. At the same time, social media platforms also allow for corrections and dialog about content to occur quickly and transparently. Moreover, information may or may not be beneficial, but the ability to understand if the information is credible and ways in which to improve critical thinking and appraisal skills of social media users should be a priority of research and practice as well as codes of conduct for posting information.

### Limitations

The most important limitation of our study is that we collected data from channels that change rapidly, at only two points in time. Consequently, the posts that were selected in our review may not be reflective of what is posted at another time. The collection of data at time points separated by 9 months may give better insights of the evolution of posts over time. However, as the content posted on social media happens constantly, data collected over time points may yield different results. Particularly on Twitter, our results are likely to be different depending on the time of the search. For example, at Time 1, we observed that 35.2% of the posts were based on the lyrics of a song, which are likely to be ephemeral. At Time 2, only 16.0% of the posts referred to that song.

The search strategy may have resulted in posts being missed. We limited the search to two search terms (“genetic” and “smoking”) and the first hundred posts, which may not have captured all the relevant posts. However, the results obtained in our search provide a reasonable perspective of what someone interested in smoking and genetics would find on the topic on YouTube, Facebook, and Twitter. Facebook, as a relatively closed system, did not allow an in-depth look at the posts of users.

The limits on the three selected social media may influence the obtained results. Other social media such as health forums might lead to different results.

### Implications for Practice and Research

This study focused on the availability of information on genetics and smoking and serves as a baseline measure from September 2012 and May 2013. Given the growing use of social media for health purposes, there is a need to monitor this situation over time to avoid the dispersion of false information. The topic of genetics and smoking is not currently widely discussed on the three social media platforms chosen. However, this is expected to change due to growing concerns about genetics in other media such as newspapers. This study did not provide any information on the profile of the viewers (eg, smokers or nonsmokers) or the use of that information (eg, subsequent change in smoking behavior). A future study assessing the habits and the characteristics of the population looking for health information (eg, general population, patients, and health professionals) and more specifically, information about genetics and smoking, will be needed. Moreover, a better overview of the users’ understanding of the displayed information will be of high importance. Also, from the scientific point of view, the concept of “genetic information” needs to be broadened towards “genome-based information”, taking into account emerging knowledge from the whole “omics” field including epigenomics and the interaction of genomics and environment, such as in the case of smoking.

This study suggests that most of the information about genetics and smoking available on social media referred to scientific publications displayed by different kind of publishers (research center, news, and medical news). Increasing access to such information might improve the health and the genomic literacy of the population and, therefore, enhance smoking prevention and cessation.

## Abbreviations

CHRNA3: Neuronal Acetylcholine Receptor subunit Alpha-3
COPD: Chronic Obstructive Pulmonary disease
EAPM: European Alliance for Personalised Medicine
ITFoM: IT Future of Medicine
PHGEN: Public Health Genomics
